# 4-Aza­niumyl-2,2,6,6-tetra­methyl­piperidin-1-ium dinitrate

**DOI:** 10.1107/S1600536814009787

**Published:** 2014-05-10

**Authors:** Hammouda Chebbi, Ridha Ben Smail, Mohamed Faouzi Zid

**Affiliations:** a Institut Préparatoire aux Etudes d’Ingénieurs de Monastir, Avenue Ibn-El-Jazzar, 5019 Monastir, Tunisia; b Laboratoire de Matériaux et Cristallochimie, Faculté des Sciences de Tunis, 2092 El Manar II, Tunis, Tunisia; c Institut Préparatoire aux Etudes d’Ingénieurs de Nabeul, Campus Universitaire Mrazka, 8000 Nabeul, Tunisia

## Abstract

In the crystal structure of the title salt, C_9_H_22_N_2_^2+^·2NO_3_^−^, the piperidine ring of the dication adopts a chair conformation and the orientation of the C—NH_3_ bond is equatorial. The ions are linked by normal and bifurcated N—H⋯O hydrogen bonds in *R*_2_^2^(6), two *R*_4_^2^(8) and *R*_3_^4^(14) graf-set motifs, generating a three-dimensional network.

## Related literature   

For related structures, see: Chebbi & Driss (2001 ▶); El Glaoui, Mrad, Jenneau & Ben Nasr (2010 ▶); Mrad *et al.* (2009 ▶); Huang & Deng (2007 ▶). For hydrogen bonding and graph-set motifs, see: Jeffrey (1997 ▶); Bernstein *et al.* (1995 ▶); Etter *et al.* (1990 ▶). For ring-puckering parameters, see: Cremer & Pople (1975 ▶); Spek (2009 ▶).
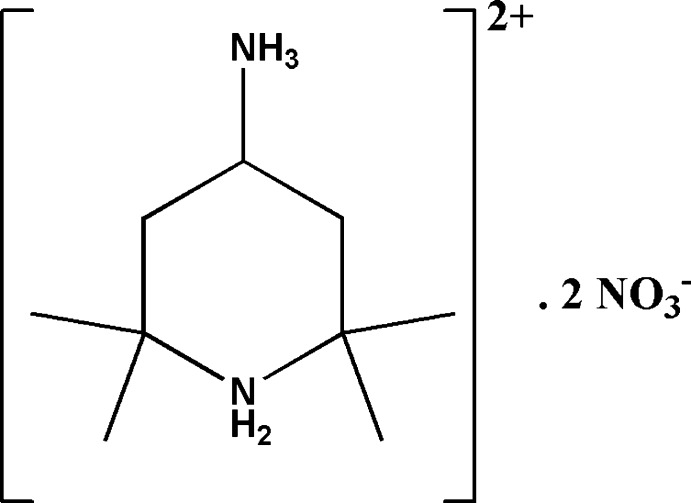


## Experimental   

### 

#### Crystal data  

C_9_H_22_N_2_^2+^·2NO_3_^−^
*M* *_r_* = 282.31
Monoclinic, 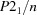

*a* = 10.367 (2) Å
*b* = 11.054 (1) Å
*c* = 13.167 (2) Å
β = 112.45 (2)°
*V* = 1394.5 (4) Å^3^
*Z* = 4
Mo *K*α radiation
μ = 0.11 mm^−1^
*T* = 298 K
0.45 × 0.30 × 0.25 mm

#### Data collection  

Enraf–Nonius CAD-4 diffractometer
Absorption correction: ψ scan (North *et al.*, 1968 ▶) *T*_min_ = 0.860, *T*_max_ = 0.978
2849 measured reflections
2731 independent reflections
1908 reflections with *I* > 2σ(*I*)
*R*_int_ = 0.017
2 standard reflections every 120 min intensity decay: 1.0%

#### Refinement  

*R*[*F* ^2^ > 2σ(*F* ^2^)] = 0.044
*wR*(*F* ^2^) = 0.121
*S* = 1.05
2731 reflections
261 parameters
All H-atom parameters refined
Δρ_max_ = 0.24 e Å^−3^
Δρ_min_ = −0.15 e Å^−3^

### 

Data collection: *CAD-4 EXPRESS* (Duisenberg, 1992 ▶; Macíček & Yordanov, 1992 ▶); cell refinement: *CAD-4 EXPRESS*; data reduction: *MolEN* (Fair, 1990 ▶); program(s) used to solve structure: *SHELXS97* (Sheldrick, 2008 ▶); program(s) used to refine structure: *SHELXL97* (Sheldrick, 2008 ▶); molecular graphics: *DIAMOND* (Brandenburg, 2001 ▶); software used to prepare material for publication: *WinGX* (Farrugia, 2012 ▶) and *publCIF* (Westrip, 2010 ▶).

## Supplementary Material

Crystal structure: contains datablock(s) I, global. DOI: 10.1107/S1600536814009787/nc2324sup1.cif

Structure factors: contains datablock(s) I. DOI: 10.1107/S1600536814009787/nc2324Isup2.hkl

Supporting information file. DOI: 10.1107/S1600536814009787/nc2324Isup3.cml

CCDC reference: 1000438

Additional supporting information:  crystallographic information; 3D view; checkCIF report

## Figures and Tables

**Table 1:** Hydrogen-bond geometry (Å, °)

*D*—H⋯*A*	*D*—H	H⋯*A*	*D*⋯*A*	*D*—H⋯*A*
N1—H1*A*⋯O1^i^	0.93 (2)	1.99 (2)	2.868 (2)	156.9 (19)
N1—H1*B*⋯O1^ii^	0.87 (2)	1.97 (2)	2.772 (2)	152.9 (19)
N2—H2*A*⋯O4	0.90 (3)	2.24 (3)	2.964 (3)	137 (2)
N2—H2*A*⋯O2^iii^	0.90 (3)	2.48 (3)	3.034 (3)	120 (2)
N2—H2*B*⋯O4^iii^	0.93 (3)	2.03 (3)	2.928 (3)	161 (2)
N2—H2*B*⋯O3^iii^	0.93 (3)	2.59 (3)	3.030 (3)	109 (2)
N2—H2*C*⋯O5^i^	0.88 (3)	2.03 (3)	2.910 (3)	172 (2)

Symmetry codes: (i) 
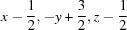
; (ii) 
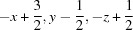
; (iii) 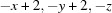.

**Fig. 1.**
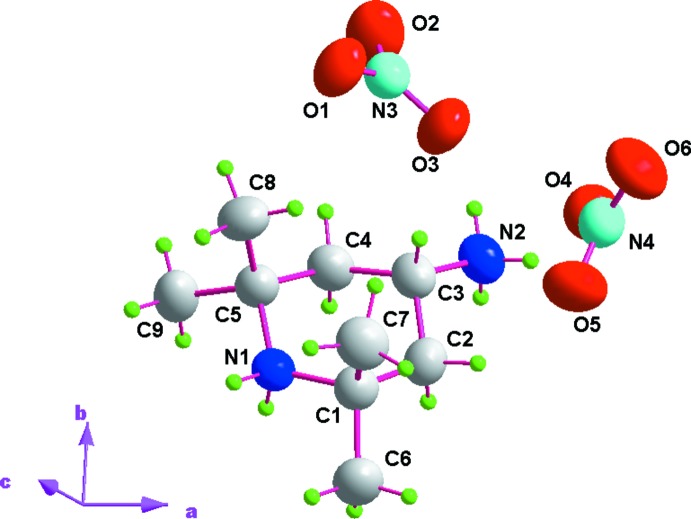
Asymmetric unit of the title compound with the atom numbering scheme. Displacement ellipsoids for non-H atoms are presented at the 50% probability level. H atoms are shown as sticks.

**Fig. 2.**
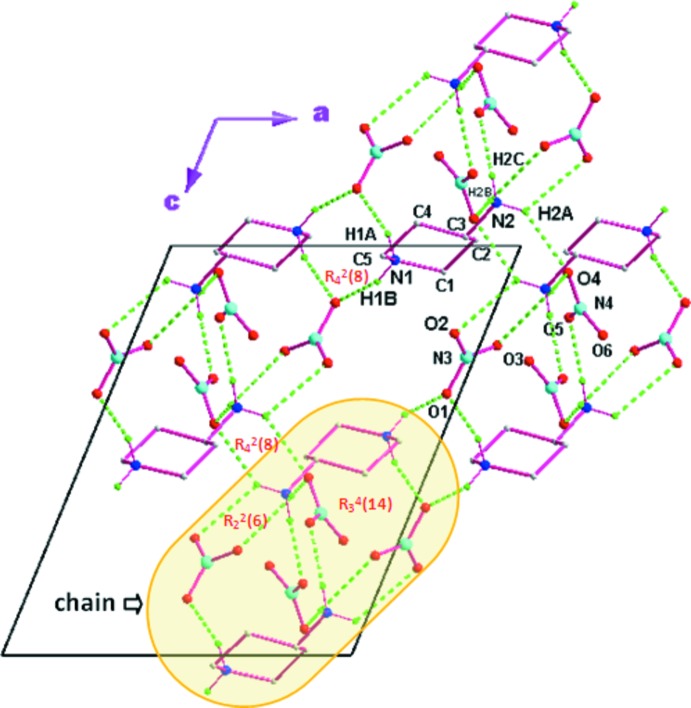
Crystal structure of the title compound with view along the b axis, showing the formation of two sets of R42(8) hydrogen-bonding motifs. Hydrogen bonds are represented by dashed lines. H atoms not involved in hydrogen bonding and –CH3 groups of 4-azaniumyl-2,2,6,6-tetramethylpiperidin-1-ium dication have been omitted for clarity.

**Fig. 3.**
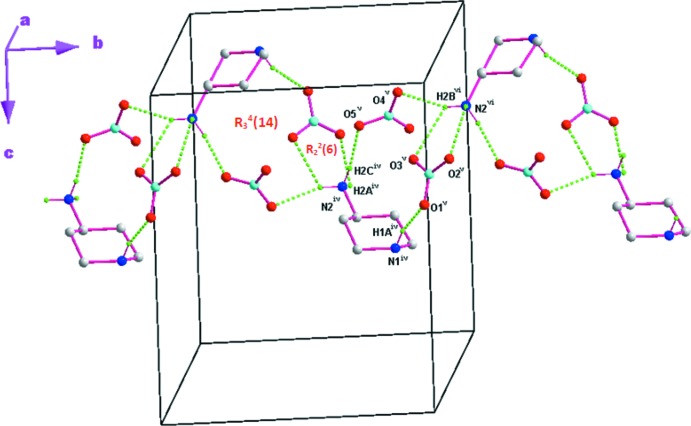
A perspective view of one chain of the title compound, showing R22(6) and R34(14) rings along [010] direction. Hydrogen bonds are represented by dashed lines. H atoms not involved in hydrogen bonding and –CH3 groups of 4-azaniumyl-2,2,6,6-tetramethylpiperidin-1-ium dication have been omitted for clarity. Symmetry codes: (iv) x - 1/2, -y + 3/2, z + 1/2; (v) x - 1, y, z; (vi) -x + 1, -y + 2, -z.

## supplementary crystallographic information

### 1. Comment

The title compound, C_9_H_22_N_2_^2+^·2NO_3_^-^, was synthesized unexpectedly from 4-amino-2,2,6,6-tetramethylpiperidine, bismuth(III) nitrate pentahydrate and nitric acid. We report in this paper it's structure; its homologues obtained with chlorate, phosphate and tetrachlorozincate anions has been described previously (Huang & Deng, 2007; Mrad *et al.*, 2009; El Glaoui *et al.*, 2010).

The asymmetric unit of the title compound contains one 4-azaniumyl-2,2,6,6-tetramethylpiperidin-1-ium dication and two nitrate anions (Fig. 1) with all atoms are located on general Wykoff position 4 e.

The piperidine ring adopts a chair conformation, with puckering parameters (calculated with *PLATON* (Spek, 2009)): Q = 0.535 Å, Θ = 6.63 ° and Φ = 205.565 ° (Cremer & Pople, 1975). This conformation has also been noticed in other 4-azaniumyl-2,2,6,6-tetramethylpiperidin-1-ium salts (Chebbi & Driss, 2001; Huang & Deng, 2007; Mrad *et al.*, 2009; El Glaoui *et al.*, 2010).

The three-dimensional extensive hydrogen-bonding network is built and linked through moderate hydrogen-bond interactions (Table 1) (Jeffrey, 1997) between the NH_3_ and NH_2_ groups of the dications and the nitrate anions, located in the vicinity of the protonated amine groups. Each organic entity is bounded to six different nitrate anions through seven N—H···O hydrogen bonds (Fig. 2). Indeed, N1—H1A···O1, N2—H2C···O5, N2—H2A···O2 and bifurcated N2—H2B···O3(O4) hydrogen bonds (Table 1) link dications and anions into chains along [010] direction, which generate *R*_3_^4^(14) and *R*_2_^2^(6) ring motifs (Etter *et al.*, 1990; Bernstein, *et al.*, 1995) (Fig. 3). These chains are interconnected by N1—H1B···O1 and N2—H2A···O4 hydrogen bonds (Table 1),which generate two sets of *R*_4_^2^(8) ring motifs (Fig. 2). This arrangement results in the formation of a complicated three-dimensional network.

### 2. Experimental

The title compound was prepared by dissolving 0.096 mmol (0.36 g) of bismuth(III) nitrate pentahydrate in 20 ml of distilled water; 0.096 mmol (0.15 g) of 4-amino-2,2,6,6-tetramethylpiperidine in 15 ml of ethanol (96%) and 1 ml of concentred nitric acid were then added. The mixture was stirred for 20 minutes and the solution is allowed to stand at room temperature. Dark brown crystals were obtained after 5 days of slow evaporation of the solvent. The X-ray analysis proves that the trivalent bismuth is not part of the structure and that the obtained phase is C_9_H_22_N_2_^2+^·2NO_3_^-^.

### 3. Refinement

All non-H atoms were refined with anisotropic atomic displacement parameters. All H atoms were located in a Fourier map and were refined isotropically.

### Crystal data

**Table:**

C_9_H_22_N_2_^2+^·2NO_3_^−^	*F*(000) = 608
*M**_r_* = 282.31	*D*_x_ = 1.345 Mg m^−^^3^
Monoclinic, *P*2_1_/*n*	Mo *K*α radiation, λ = 0.71073 Å
Hall symbol: -P 2yn	Cell parameters from 25 reflections
*a* = 10.367 (2) Å	θ = 10–15°
*b* = 11.054 (1) Å	µ = 0.11 mm^−^^1^
*c* = 13.167 (2) Å	*T* = 298 K
β = 112.45 (2)°	Prism, dark brown
*V* = 1394.5 (4) Å^3^	0.45 × 0.30 × 0.25 mm
*Z* = 4	

### Data collection

**Table:**

Enraf–Nonius CAD-4 diffractometer	1908 reflections with *I* > 2σ(*I*)
Radiation source: fine-focus sealed tube	*R*_int_ = 0.017
Graphite monochromator	θ_max_ = 26.0°, θ_min_ = 2.5°
ω/2θ scans	*h* = −11→12
Absorption correction: ψ scan (North *et al.*, 1968)	*k* = −13→0
*T*_min_ = 0.860, *T*_max_ = 0.978	*l* = −16→0
2849 measured reflections	2 standard reflections every 120 min
2731 independent reflections	intensity decay: 1.0%

### Refinement

**Table:**

Refinement on *F*^2^	Secondary atom site location: difference Fourier map
Least-squares matrix: full	Hydrogen site location: inferred from neighbouring sites
*R*[*F*^2^ > 2σ(*F*^2^)] = 0.044	All H-atom parameters refined
*wR*(*F*^2^) = 0.121	*w* = 1/[σ^2^(*F*_o_^2^) + (0.0508*P*)^2^ + 0.3472*P*] where *P* = (*F*_o_^2^ + 2*F*_c_^2^)/3
*S* = 1.05	(Δ/σ)_max_ = 0.001
2731 reflections	Δρ_max_ = 0.24 e Å^−^^3^
261 parameters	Δρ_min_ = −0.15 e Å^−^^3^
0 restraints	Extinction correction: *SHELXL97* (Sheldrick, 2008), Fc^*^=kFc[1+0.001xFc^2^λ^3^/sin(2θ)]^-1/4^
Primary atom site location: structure-invariant direct methods	Extinction coefficient: 0.022 (3)

### Special details

**Table:**

Geometry. All e.s.d.'s (except the e.s.d. in the dihedral angle between two l.s. planes) are estimated using the full covariance matrix. The cell e.s.d.'s are taken into account individually in the estimation of e.s.d.'s in distances, angles and torsion angles; correlations between e.s.d.'s in cell parameters are only used when they are defined by crystal symmetry. An approximate (isotropic) treatment of cell e.s.d.'s is used for estimating e.s.d.'s involving l.s. planes.
Refinement. Refinement of *F*^2^ against ALL reflections. The weighted *R*-factor *wR* and goodness of fit *S* are based on *F*^2^, conventional *R*-factors *R* are based on *F*, with *F* set to zero for negative *F*^2^. The threshold expression of *F*^2^ > σ(*F*^2^) is used only for calculating *R*-factors(gt) *etc*. and is not relevant to the choice of reflections for refinement. *R*-factors based on *F*^2^ are statistically about twice as large as those based on *F*, and *R*- factors based on ALL data will be even larger.

### Fractional atomic coordinates and isotropic or equivalent isotropic displacement parameters (Å^2^)

**Table:**

	*x*	*y*	*z*	*U*_iso_*/*U*_eq_	
N1	0.65815 (16)	0.64785 (14)	0.03529 (14)	0.0327 (4)	
H1A	0.607 (2)	0.614 (2)	−0.0322 (19)	0.051 (6)*	
H1B	0.636 (2)	0.6145 (19)	0.0858 (17)	0.041 (6)*	
N2	0.8793 (2)	0.8680 (2)	−0.10476 (17)	0.0459 (5)	
H2A	0.972 (3)	0.854 (2)	−0.085 (2)	0.072 (8)*	
H2B	0.857 (3)	0.950 (3)	−0.109 (2)	0.076 (9)*	
H2C	0.838 (3)	0.834 (2)	−0.170 (2)	0.059 (7)*	
C1	0.80955 (19)	0.61008 (17)	0.06283 (15)	0.0356 (5)	
C2	0.8620 (2)	0.67784 (19)	−0.01499 (17)	0.0392 (5)	
H2D	0.959 (2)	0.666 (2)	0.0075 (17)	0.050 (6)*	
H2E	0.820 (2)	0.6457 (19)	−0.0867 (18)	0.047 (6)*	
C3	0.8315 (2)	0.81240 (18)	−0.02154 (16)	0.0360 (5)	
H3	0.881 (2)	0.8515 (17)	0.0414 (16)	0.034 (5)*	
C4	0.6767 (2)	0.8365 (2)	−0.05477 (18)	0.0396 (5)	
H4A	0.627 (2)	0.8029 (18)	−0.1287 (18)	0.044 (6)*	
H4B	0.660 (2)	0.921 (2)	−0.0601 (17)	0.046 (6)*	
C5	0.6175 (2)	0.78055 (17)	0.02436 (16)	0.0366 (5)	
C6	0.8059 (3)	0.4739 (2)	0.0425 (3)	0.0533 (6)	
H6A	0.759 (3)	0.455 (2)	−0.032 (2)	0.067 (8)*	
H6B	0.772 (3)	0.431 (2)	0.091 (2)	0.068 (8)*	
H6C	0.899 (3)	0.449 (3)	0.060 (2)	0.082 (9)*	
C7	0.8987 (3)	0.6347 (3)	0.18384 (18)	0.0512 (6)	
H7A	0.848 (3)	0.609 (3)	0.229 (2)	0.088 (9)*	
H7B	0.922 (3)	0.721 (3)	0.201 (2)	0.075 (8)*	
H7C	0.983 (3)	0.587 (2)	0.201 (2)	0.072 (8)*	
C8	0.6690 (3)	0.8427 (2)	0.1367 (2)	0.0530 (6)	
H8A	0.625 (3)	0.921 (3)	0.127 (2)	0.075 (8)*	
H8B	0.774 (3)	0.854 (2)	0.1702 (19)	0.062 (7)*	
H8C	0.640 (2)	0.798 (2)	0.186 (2)	0.059 (7)*	
C9	0.4579 (2)	0.7821 (2)	−0.0251 (2)	0.0513 (6)	
H9A	0.430 (3)	0.867 (3)	−0.038 (2)	0.072 (8)*	
H9B	0.425 (2)	0.742 (2)	0.0249 (19)	0.052 (6)*	
H9C	0.423 (3)	0.732 (2)	−0.099 (2)	0.068 (7)*	
N3	0.97658 (17)	1.01245 (16)	0.27175 (13)	0.0414 (4)	
O1	0.96306 (16)	0.99288 (15)	0.36111 (11)	0.0581 (5)	
O2	0.9133 (2)	1.09726 (19)	0.21402 (16)	0.0806 (6)	
O3	1.0499 (2)	0.94507 (17)	0.24364 (15)	0.0735 (6)	
N4	1.24475 (18)	0.83006 (17)	0.15347 (14)	0.0452 (4)	
O4	1.16626 (17)	0.87793 (16)	0.06514 (12)	0.0578 (5)	
O5	1.2237 (2)	0.72440 (15)	0.17517 (14)	0.0692 (5)	
O6	1.34062 (19)	0.88941 (19)	0.21907 (14)	0.0779 (6)	

### Atomic displacement parameters (Å^2^)

**Table:**

	*U*^11^	*U*^22^	*U*^33^	*U*^12^	*U*^13^	*U*^23^
N1	0.0349 (9)	0.0339 (9)	0.0300 (8)	−0.0027 (7)	0.0131 (7)	0.0000 (7)
N2	0.0492 (12)	0.0511 (13)	0.0432 (11)	−0.0107 (10)	0.0242 (9)	0.0002 (9)
C1	0.0318 (10)	0.0372 (11)	0.0366 (10)	0.0014 (8)	0.0115 (8)	0.0010 (8)
C2	0.0348 (11)	0.0467 (12)	0.0383 (11)	0.0011 (9)	0.0165 (9)	−0.0018 (9)
C3	0.0367 (10)	0.0423 (11)	0.0298 (10)	−0.0076 (9)	0.0136 (8)	−0.0026 (9)
C4	0.0411 (11)	0.0366 (12)	0.0404 (11)	0.0002 (9)	0.0149 (9)	0.0060 (9)
C5	0.0392 (11)	0.0316 (10)	0.0409 (11)	0.0008 (8)	0.0176 (9)	0.0004 (8)
C6	0.0536 (15)	0.0400 (13)	0.0697 (17)	0.0061 (11)	0.0273 (14)	0.0030 (12)
C7	0.0458 (13)	0.0594 (16)	0.0384 (12)	0.0019 (12)	0.0049 (10)	0.0069 (11)
C8	0.0714 (17)	0.0443 (14)	0.0518 (14)	−0.0047 (12)	0.0332 (13)	−0.0113 (11)
C9	0.0405 (12)	0.0452 (14)	0.0740 (17)	0.0067 (11)	0.0284 (12)	0.0114 (13)
N3	0.0442 (10)	0.0464 (10)	0.0364 (9)	−0.0044 (8)	0.0184 (8)	−0.0020 (8)
O1	0.0696 (11)	0.0758 (12)	0.0380 (8)	0.0214 (9)	0.0308 (8)	0.0116 (8)
O2	0.0791 (13)	0.0862 (14)	0.0787 (13)	0.0209 (11)	0.0326 (10)	0.0404 (11)
O3	0.0933 (13)	0.0714 (12)	0.0814 (13)	0.0112 (10)	0.0620 (11)	−0.0097 (10)
N4	0.0438 (10)	0.0528 (12)	0.0404 (10)	0.0018 (9)	0.0178 (8)	0.0005 (9)
O4	0.0591 (10)	0.0670 (11)	0.0409 (8)	0.0074 (8)	0.0121 (7)	0.0089 (8)
O5	0.0950 (14)	0.0465 (10)	0.0616 (11)	−0.0037 (9)	0.0247 (10)	0.0053 (8)
O6	0.0676 (12)	0.0939 (15)	0.0564 (10)	−0.0306 (11)	0.0061 (9)	−0.0065 (10)

### Geometric parameters (Å, º)

**Table:**

N1—C5	1.518 (2)	C5—C8	1.530 (3)
N1—C1	1.528 (2)	C6—H6A	0.94 (3)
N1—H1A	0.93 (2)	C6—H6B	0.97 (3)
N1—H1B	0.87 (2)	C6—H6C	0.95 (3)
N2—C3	1.496 (2)	C7—H7A	0.97 (3)
N2—H2A	0.90 (3)	C7—H7B	0.99 (3)
N2—H2B	0.93 (3)	C7—H7C	0.97 (3)
N2—H2C	0.88 (3)	C8—H8A	0.97 (3)
C1—C2	1.527 (3)	C8—H8B	1.01 (2)
C1—C6	1.527 (3)	C8—H8C	0.95 (3)
C1—C7	1.530 (3)	C9—H9A	0.98 (3)
C2—C3	1.516 (3)	C9—H9B	0.95 (2)
C2—H2D	0.94 (2)	C9—H9C	1.05 (3)
C2—H2E	0.95 (2)	N3—O3	1.219 (2)
C3—C4	1.517 (3)	N3—O2	1.227 (2)
C3—H3	0.90 (2)	N3—O1	1.256 (2)
C4—C5	1.527 (3)	N4—O6	1.228 (2)
C4—H4A	0.98 (2)	N4—O5	1.241 (2)
C4—H4B	0.95 (2)	N4—O4	1.254 (2)
C5—C9	1.530 (3)		
			
C5—N1—C1	120.63 (14)	N1—C5—C4	106.67 (15)
C5—N1—H1A	105.5 (14)	N1—C5—C9	105.52 (16)
C1—N1—H1A	106.3 (14)	C4—C5—C9	110.84 (17)
C5—N1—H1B	109.7 (14)	N1—C5—C8	111.14 (17)
C1—N1—H1B	104.7 (14)	C4—C5—C8	113.24 (18)
H1A—N1—H1B	109.7 (19)	C9—C5—C8	109.1 (2)
C3—N2—H2A	109.4 (16)	C1—C6—H6A	111.5 (16)
C3—N2—H2B	107.5 (17)	C1—C6—H6B	111.3 (15)
H2A—N2—H2B	113 (2)	H6A—C6—H6B	114 (2)
C3—N2—H2C	111.5 (16)	C1—C6—H6C	107.1 (17)
H2A—N2—H2C	106 (2)	H6A—C6—H6C	105 (2)
H2B—N2—H2C	109 (2)	H6B—C6—H6C	107 (2)
C2—C1—C6	110.92 (18)	C1—C7—H7A	109.7 (17)
C2—C1—N1	107.66 (15)	C1—C7—H7B	113.9 (15)
C6—C1—N1	105.86 (17)	H7A—C7—H7B	106 (2)
C2—C1—C7	112.54 (18)	C1—C7—H7C	106.3 (15)
C6—C1—C7	108.8 (2)	H7A—C7—H7C	110 (2)
N1—C1—C7	110.82 (17)	H7B—C7—H7C	111 (2)
C3—C2—C1	113.53 (16)	C5—C8—H8A	107.8 (15)
C3—C2—H2D	109.4 (14)	C5—C8—H8B	113.4 (13)
C1—C2—H2D	109.2 (13)	H8A—C8—H8B	109 (2)
C3—C2—H2E	107.8 (13)	C5—C8—H8C	110.5 (14)
C1—C2—H2E	109.8 (13)	H8A—C8—H8C	107 (2)
H2D—C2—H2E	107.0 (18)	H8B—C8—H8C	109 (2)
N2—C3—C2	108.91 (17)	C5—C9—H9A	106.6 (15)
N2—C3—C4	109.06 (17)	C5—C9—H9B	108.1 (14)
C2—C3—C4	111.33 (17)	H9A—C9—H9B	114 (2)
N2—C3—H3	104.1 (12)	C5—C9—H9C	108.5 (14)
C2—C3—H3	112.6 (12)	H9A—C9—H9C	112 (2)
C4—C3—H3	110.5 (12)	H9B—C9—H9C	107.9 (19)
C3—C4—C5	112.84 (16)	O3—N3—O2	121.79 (19)
C3—C4—H4A	108.2 (12)	O3—N3—O1	119.03 (18)
C5—C4—H4A	109.2 (12)	O2—N3—O1	119.16 (18)
C3—C4—H4B	110.0 (13)	O6—N4—O5	120.45 (19)
C5—C4—H4B	109.7 (13)	O6—N4—O4	119.4 (2)
H4A—C4—H4B	106.7 (17)	O5—N4—O4	120.12 (19)

### Hydrogen-bond geometry (Å, º)

**Table:**

*D*—H···*A*	*D*—H	H···*A*	*D*···*A*	*D*—H···*A*
N1—H1*A*···O1^i^	0.93 (2)	1.99 (2)	2.868 (2)	156.9 (19)
N1—H1*B*···O1^ii^	0.87 (2)	1.97 (2)	2.772 (2)	152.9 (19)
N2—H2*A*···O4	0.90 (3)	2.24 (3)	2.964 (3)	137 (2)
N2—H2*A*···O2^iii^	0.90 (3)	2.48 (3)	3.034 (3)	120 (2)
N2—H2*B*···O4^iii^	0.93 (3)	2.03 (3)	2.928 (3)	161 (2)
N2—H2*B*···O3^iii^	0.93 (3)	2.59 (3)	3.030 (3)	109 (2)
N2—H2*C*···O5^i^	0.88 (3)	2.03 (3)	2.910 (3)	172 (2)

Symmetry codes: (i) *x*−1/2, −*y*+3/2, *z*−1/2; (ii) −*x*+3/2, *y*−1/2, −*z*+1/2; (iii) −*x*+2, −*y*+2, −*z*.
